# Physicians’ pharmacogenomics information needs and seeking behavior: a study with case vignettes

**DOI:** 10.1186/s12911-017-0510-9

**Published:** 2017-08-01

**Authors:** Bret S. E. Heale, Aly Khalifa, Bryan L. Stone, Scott Nelson, Guilherme Del Fiol

**Affiliations:** 1 Department of Biomedical Informatics, University of Utah, 421 Wakara Way, Salt Lake City, UT 84108 USA; 2 Intermountain Healthcare, West Valley, UT USA; 3 Department of Pediatrics, University of Utah, Salt Lake City, UT USA; 4 Vanderbilt University Medical Center, Nashville, TN USA

**Keywords:** Physician information needs assessment, Pharmacogenomics, Information seeking behavior, Access to information

## Abstract

**Background:**

Genetic testing, especially in pharmacogenomics, can have a major impact on patient care. However, most physicians do not feel that they have sufficient knowledge to apply pharmacogenomics to patient care. Online information resources can help address this gap. We investigated physicians’ pharmacogenomics information needs and information-seeking behavior, in order to guide the design of pharmacogenomics information resources that effectively meet clinical information needs.

**Methods:**

We performed a formative, mixed-method assessment of physicians’ information-seeking process in three pharmacogenomics case vignettes. Interactions of 6 physicians’ with online pharmacogenomics resources were recorded, transcribed, and analyzed for prominent themes. Quantitative data included information-seeking duration, page navigations, and number of searches entered.

**Results:**

We found that participants searched an average of 8 min per case vignette, spent less than 30 s reviewing specific content, and rarely refined search terms. Participants’ information needs included a need for clinically meaningful descriptions of test interpretations, a molecular basis for the clinical effect of drug variation, information on the logistics of carrying out a genetic test (including questions related to cost, availability, test turn-around time, insurance coverage, and accessibility of expert support).Also, participants sought alternative therapies that would not require genetic testing.

**Conclusion:**

This study of pharmacogenomics information-seeking behavior indicates that content to support their information needs is dispersed and hard to find. Our results reveal a set of themes that information resources can use to help physicians find and apply pharmacogenomics information to the care of their patients.

**Electronic supplementary material:**

The online version of this article (doi:10.1186/s12911-017-0510-9) contains supplementary material, which is available to authorized users.

## Background

As a component of personalized medicine, pharmacogenomics testing can reduce occurrences of complications due to adverse drug events, improve quality of patient care, reduce financial costs [1–6], and lead to improvements in patient treatment adherence [7, 8]. Highlighting the critical importance of pharmacogenomics in personalized medicine, adverse drug events were reported in 2014 to cause death or other serious outcomes in 807, 270 people in the US [9]. As an example of pharmacogenomics, in asthma treatment with beta-adrenergic receptor agonists, studies recommend that patients with specific genetic variants be given an alternative treatment [2, 5, 6, 10]. In 2010, this could have affected treatment decisions for 12 million Americans suffering asthma who were expected to experience acute symptoms [11].

The evidence regarding medications with known pharmacogenomics implications is rapidly increasing, ranging from expert opinion supported by the science to an extent (weaker) to meta-analyses of randomized clinical trials (stronger), but there are significant barriers to the optimal adoption of pharmacogenomics information in routine patient care decisions. Compared to other medical domains, physicians have a self-identified lack of pharmacogenomics knowledge and low ‘self-efficacy’ in pharmacogenomics testing. Only 10% of physicians nationwide, based on a 2008 National Survey [12], feel that they have adequate understanding of pharmacogenomics tests, while 98% believe pharmacogenomics testing will benefit their patients [12]. In response, attempts have been made to better integrate pharmacogenomics information into medical education [13–15], and several organizations promote the accessibility of pharmacogenomics knowledge [4]. However, physicians still feel inadequately informed. A more recent survey by Selkirk et al. found that 51% of physicians at the NorthShore University HealthSystem considered themselves to have no to minimal pharmacogenomics knowledge [16]. Similar results were found for ‘when and how to incorporate genomic medicine into practice.’

Knowledge gaps are a significant concern for clinicians that can be addressed through online information resources [17, 18], to improve provider decisions, patient outcomes [19–21], and time [22–25]. As a response to this need, groups such as the Clinical Pharmacogenetics Implementation Consortium (CPIC) have published guidelines on pharmacogenomics testing [26]. However, more recent reviews and research indicate that searching for pharmacogenomics information that can be applied in patient care is still quite challenging [27–31]. To help guide delivery of content from clinical pharmacogenomics resources, we investigated physicians’ information needs and information-seeking behavior when exposed to pharmacogenomics case vignettes.

## Methods

A formative, mixed-methods study was designed to capture physicians’ pharmacogenomics information needs and information-seeking behavior. The study consisted of a pre-study questionnaire of attitudes and knowledge regarding pharmacogenomics, observation of physicians’ information-seeking to address three case vignettes, a post-study questionnaire, and a post-study interview.

### Demographics of sample population

A purposive sample of six physicians, to represent various ages and levels of clinical experience, was recruited to participate in the study. Five male and one female pediatricians and internists participated as subjects. Three were 30–39 years-old, two were 40–49 years-old and one was 60–69 years-old. Years in practice ranged from 2 to 36, averaging 13.8. All practiced in the same large, urban hospital setting.

### Case vignette design

Case vignettes were designed by examining existing case vignettes (case 1 [32–34]:) and guidelines (case 1 [35]:; case 2 [6, 36, 37]:; case 3 [38, 39]:). Vignettes were iteratively refined and validated by a clinical domain expert. Each vignette consisted of a case description followed by prompts for a pharmacogenomics information search (Table 1 and Additional file 1). The overall goal was to produce a purposive sample of case vignettes that triggered a wide range of information needs and information seeking behavior. For Case 1, information regarding treatment decisions and genetic variation is easier to find and less controversial. While not a typical example of the application of pharmacogenomics, patients who display symptoms of Gaucher disease benefit from tailoring therapy based on genetic variation. Cases 2 and 3 are more typical examples of pharmacogenomics. We did not find major variation in the themes derived from cases 1, 2 and 3.

**Table 1:** Case vignette summary

Case vignette	Disease or condition	Medication focus	Problem	Patient	Main information-seeking driver
1	Gaucher’s disease	Enzyme Replacement Therapy	Hereditary risk	Prospective child of at risk parents	Parents desire to be prepared
2	Asthma	Albuterol	Worsening symptoms while on treatment	Pediatric male, no apparent environmental factors, twin sister with same problem	Father’s concern
3	Percutaneous coronary intervention	Clopidogrel	Loading dose prescription	65 –year old male, past smoker, no history of bleeding or increased clotting	Patient’s concern

### Data collection

First, subjects filled out a pre-study survey (Additional file 2) on personal demographics, internet resource experience, pharmacogenomics attitude and clinical experience. The pre-study survey contained questions from Stanek et al. [12] and Del Fiol et al. [40] to collect information on subjects’ professional background and internet experience (Additional file 2). Next, subjects read vignettes and were asked to search specific internet resources: UpToDate® (all cases) and PharmGKB (Pharmacogenomics Knowledge Base) (case 3). UpToDate is written and reviewed by expert authors who cite supporting evidence for the recommendations made in their articles. PharmGKB is authored by a Pharmacogenomics consortium and presents evidence from clinical guidelines and basic research. Subjects were asked to ‘think-aloud’ , sharing their thoughts as they sought information. Audio/computer-screen interactions were recorded while subjects searched. During the information search, BH took notes to aid the post-session interview. After Cases Two and Three, subjects filled out a brief post-study survey including open-ended questions on search satisfaction, information retrieved and unfulfilled information needs (Additional file 2).The post-survey questions were adapted from Del Fiol et al. [41] to measure satisfaction with the results of an online search and provide the participant an opportunity to provide unfettered feedback on the case vignette and information search. After Case Three, BH interviewed subjects using the audio/computer-screen recording and notes to elicit specific points of the information-seeking experience. Session time was 50 to 90 min.

### Qualitative data analysis

Think-aloud audio and post-study interviews were transcribed, de-identified, and time-stamped. The transcripts along with open-ended questions from the post-study survey were subject to content analysis, as described in Berg et al. [41] Initially, BH performed open-coding for one subject using both the audio transcripts and screen recordings. Next, BH and GDF refined the initial codes for inclusion into a study code book. For reliability and validity, BH and AK coded each subject independently and iteratively reconciled disagreements through consensus. Overall, there were 42 coded information needs (Additional file 3). Last, BH and AK analyzed information needs by merging similar codes into higher level themes. The set of themes was refined through discussions among BH, GDF, and BS.

### Theoretical framework guiding data analysis

Coding was guided by an information-seeking model called ‘Berry-Picking’ , which is applicable in situations where the information seeker is inexpert in the subject of interest, such as primary care physicians seeking new pharmacogenomics information [42]. According to ‘Berry-Picking’ , in order to fulfill an information need, the subject exercises strategies to satisfy the need, seeking locations of information that are compared to ‘berry’ patches. As the subject finds relevant ‘berries’ , the understanding of the problem improves, leading to more refined and specific information-seeking strategies. Each iteration of information-seeking leads to progressively more sophisticated inquiries.

### Quantitative data analysis

To analyze information-seeking behavior we computed median, average, range, and standard deviation for the following: time spent by each physician on information-seeking, time between navigational actions (i.e., clicking links/tabs), number of navigational actions, and number of searches entered. The time between navigational actions represents the time subjects spent considering specific content before leaving to other content.

## Results

### Pre-study survey - pharmacogenomics experience and attitudes

Subjects indicated internet and UpToDate® search proficiency, but were not familiar with PharmGKB. All subjects felt that genetics influences treatment response. Only one of six felt adequately informed about genetic testing. One subject had education in pharmacogenomics. Colleagues were a source of routine pharmacogenomics information for five subjects. Additionally, two subjects indicated that the internet was a routine source of pharmacogenomics information, and these subjects had previous pharmacogenomics testing experience. All subjects indicated that private, state, and federal health insurers should provide full coverage for pharmacogenomics testing in some cases.

### Themes

Qualitative analysis revealed 11 themes that merged into 6 categories (Table 2).

**Table 2:** Information needs categories and themes

Category	Theme
Is there an alternative therapy that obviates the need for genetic testing?	Alternative therapy options
Clear, reliable guidance on genetic testing: when and how	Specific, actionable, clinical guidance from authoritative sources Guidance on optimal approach to genetic testing Logistics of testing
How often might genetic testing be indicated?	Prevalence of genetic variation Indications for genetic testing
How important is genetic testing to care of my patient and what is the evidence?	Clinical impact of genetic testing Practice changing evidence
Help in understanding genetic effects	Role of genetics in the manifestation of the disease Understanding general molecular effect of genetic variant
Aid in searching for information	Help with search terms

### Alternative therapy options

Subjects sought evidence that alternative treatments could be used effectively and safely without requiring a genetic test, or that such an alternative therapy was not available.

For example, subject 5 sought a recommendation “…*telling you to use another option in him. Or, does [the patient] have a particular contraindication against alternate therapy [that] I’m supposed to pick up on, instead of doing any testing on him*.” Similarly, a different subject expressed that “*ideally what I am thinking is to maybe just change the therapy and regimen… just switch to something else*.”

### Specific, actionable, clinical guidance from authoritative sources

Subjects need an authoritative, ‘bottom-line’ recommendation. For example, subject 3 stated, *“with the authorities, with the experts, with the review of the literature, American Heart Association and the rest, where they will be saying, ‘you ought to test these patients, because you are [going to] lower your risks related to putting that stent in.’ That’s what I was looking for.”* Similarly, subject 3 was *“going for a bottom-line.”* Additionally, consult with a specialist was considered as evidenced by subjects navigating to and examining available ‘Genetic Counseling’ sections.

### Guidance on optimal approach to genetic testing

Subjects wanted to know the best genetic test to use. For example, subject 3 mentioned “*I'm not finding anything that is like a straightforward test…”*, and subject 5 stated they were “*looking for any indication that I should select a particular genetic test option*.”

They were also curious about choosing one approach over the other, evidenced by subject 6 stating *“genotyping may miss some of those loss of function alleles. And, I just find it interesting that it is a footnote because it seems rather important to me”.*

### Logistics of testing

Subjects wanted information on whether they could order a test in their system, the test turn-around time, and considered both cost and proximity of an expert. For example, subject 4 mentioned “*first I’d have to find out if a test even exists and how much it costs.*’” And subject 4 asked *“how fast can we make the genomic test actually be available?”* Getting a test result in time to make decisions was a concern. For example, subject 3 asked “*But can they turn [the genetic test] around? you would have to know, (especially) if the guy is going to have the stent the next day.”* Subject 3 wondered if the result could be achieved “*in half an hour with DOT-Blot-PCR.”*

Other cost considerations were expressed by subject 3 during the post-session interview: *“Other information that could have helped me? Knowing the prevalence of the CYP2C19 (Cytochrome P450 2C19) mutations in the general population and in subset populations, knowing if the patient had family members with known difficulty in metabolizing medications, cost of the test, availability of the test, turnaround time of the test*”. Subject 1 voiced a perception that the financial cost is high by indicating that the subject would order a test “*if this guy is rich and wants to be parted with some of his money…”* And subject 4, when prompted for thoughts on why cost matters *“if you have a test that costs $500,000 to do, then most of the time I’m going to say no, it’s not even remotely worthwhile.”* Even a ‘ballpark’ estimate would at least inform *“[cost] in the range of hundreds as opposed to thousands, as opposed to hundreds of thousands. The cost of it is so important for any new thing...”*

Subject 6 also mentioned concern regarding the proximity to a specialist *“but if you have implications to a child’s wellbeing and maybe mortality …I have the resources right here I use them…that’s sort of a bit harder if you are asking your family to drive 400 miles to a pharmacogenetic counselor.”*

### Prevalence of genetic variation

Information on prevalence of genetic variation was also sought; both for the population in general, for the patient’s ethnic group, and in the patient’s family. For example, subject 4 stated *“it doesn't really tell me what percentage of the population has an issue with CYP2c19.”* Along these lines, subject 6 stated *“…just how many individuals do I need to test before I find…individuals who either metabolize rapidly or poorly”.*

The following demonstrates subject 4’s use of family history. When asked ‘is that important?’ in relation to highlighting autosomal co-dominance during case 2, subject 4 responded *“It is important because… if there had been a pure autosomal dominant, maybe you would actually have a family history of something that would be relevant.”*

### Indications for genetic testing

Subjects sought information on the patient characteristics which indicate a benefit or imperative for testing. For example, subject 5 remarked, *“the information that I was looking for the whole time that I didn’t feel like I really found in a really concentrated way, was here are the risk factors that you as the clinician [want to] be looking for in your patient that is [going to] send you over the edge to get genetic testing.”* Subject 5 stated that they wanted *“a bulleted list that says risk factors for testing.”* Additionally, subject 3 stated *“…some instance group where you were to tell me that [my patient] needed to be tested before he had a stent put in. That’s what I was searching for.”* Also, when subject 5 was asked what they were looking for when they highlighted *“select populations”* in the CPIC guidelines, subject 5 responded *“same thing that I wanted the whole time…what is this selected patient population…”.*

### Clinical impact of genetic testing

Subjects wanted to know the clinical impact of genetic testing. Impact was indicated by the manifestation of treatment failure. For example, subject 6, after having entered in a search term containing a drug and gene name, focused on the sections of UpToDate which defined ‘resistance’ and ‘nonresponsiveness’ to treatment. When asked about this focus, subject 6 said they were *“trying to understand the relevance of the HPR* [high on-treatment platelet reactivity] *to the therapeutic intervention.”* HPR is an indication of treatment failure.

Impact was measured by the actions needed to address treatment failure. For example, in case 2 while looking at information on asthma exacerbation subject 3 remarked *“asthma exacerbation in children, that might be a good start…I'm looking for something that…oh, management criteria...”*

Also, impact was understood from the change in treatment course that could result from the genetic test. For example, if the test result interpretation is associated with a different treatment regimen, e.g. subject 3 exclaimed *“…so, they're suggesting another drug for intermediate metabolizers.*”

Further, impact was measured by the effect of ignoring genetic testing in specific situations, i.e. the effect of a genetic variation on the likelihood of treatment failure, and likelihood of severe side-effects. This was evidenced by subject 3 examining the effects of medication resistance and treatment failure, and by subject 1 remarking, *“I like this failure thing because I wonder if [genetics] might have something to do with [the severity of treatment failure]”.* Additionally, subject 2 stated *“I wanted to look at what information there was about patients with asthma and albuterol and how their genetic profile affects albuterol.”* Subject 2 went on to say *“I was hoping that I would see something like albuterol failure in asthma, or issues associated with treatment, or treatment failure in asthma, or something like that. And then when I clicked on that link there [would be] a sub-tab like genetic issues or genetic variance or something like that.”*

Finally, impact of genetic testing was measured by an explicit connection between a test result and a phenotype of the patient. Subject 6 stated, “*UpToDate suggested ah, at least based on my reading that genotypic testing is in most cases not helpful or difficult to interpret. Whereas here [Clinical Pharmacogenomics Implementation Consortium guideline] I’m seeing recommendations that are strong… based on a genotypic classification suggesting a phenotype that has relevance to your therapeutic intervention.”*

### Practice changing evidence

Subjects were looking at evidence not only for assurance that an association existed but also for assurance that the association was practice changing. Subjects looked for succinct, strongly worded statements from authoritative sources as practicing changing evidence. This idea is typified by the statement from subject 6 *“But essentially you know whenever [the] clinical literature says ‘data suggests’ or ‘maybe relevant, ’ that sort of thing, you realize that the evidence basis is still in its nascence. It may still be weak”.*

Evidence was also drawn from phenotypic explanations of manifestations of resistance or disease. For example, subject 6, while using the mouse to highlight implications for clopidogrel treatment of poor metabolizers in Table 2 of the CPIC guidelines, where it also says the level of evidence is strong, stated *“briefly reading this, this group suggests that there is benefit to looking at CYP2C19 status.”* Further, indications for treatment made subject 4 *“hopeful that guideline updates were there that directed the actual testing recommendations.”*

Additionally, evidence came in the form of evidence-based routine recommendation for testing. For example, subject 5 while browsing the results of the ARTIC (Assessment by a double Randomisation of a Conventional antiplatelet strategy versus a monitoring-guided strategy for drug-eluting stent implantation and, of Treatment Interruption versus Continuation 1 year after stenting)-Monitoring trial read-aloud *“do not recommend routine testing.”* Also, subject 3 noted *“it is not completely clear in my mind yet about the evidence for doing the testing for the variants…how standard that is and how clear that is.”* Finally, subject 1 concluded *“the summary did not have any recommendations. It had a summary of the data but the data was too far removed from me actually being able to take one step or another”,* and subject 6 stated *“I liked that it was, for the clinical question we had, simpler and more succinct than UpToDate. But it seemed that…they [Clinical Pharmacogenomics Implementation Consortium guideline] were more confident in the evidence base than the final summary recommendation in UpToDate.”*

Interestingly, subject 1 commented that it was important to know how often the patient’s specific demographic was included in the evidence for genetic testing interpretations, *“if they are unrepresented in the studies, then how am I going to know how …to interpret this. I am liable to come back with something that says ‘we do not have enough information’.”*

Finally, evidence was sought from randomized controlled trials supporting the hypothesis that a specific genetic test improves patient outcomes compared to alternatives. For example, subject 2 highlighted and read aloud the description of the RAPID GENE Study.

### Role of genetics in the manifestation of the disease

In looking at drug-disease pairs and genetics, subjects sought to determine if genetics had been associated with the symptoms and prognosis of the disease. For example, subject 3 stated “*I would want to know about the genetic variant effects that are associated with worsening asthma symptoms*” and was looking for a statement that “*might have said that there was a genetic predisposition to worsening symptoms*.”

### Understanding general molecular effect of genetic variant

Subjects needed to understand the effect of molecular changes on protein activity. For example, subject 6 remarked *“…trying to recall my molecular biology…and ah, what is a missense mutation.”*

### Help with search terms

Subjects sought guidance on constructing search terms, such as the correct spelling of a gene name, useful synonyms, or medication specific detail. Key search terms were considered gateways to satisfying information needs. For example, subject 4 copied and pasted the CYP2C19 as a search term. Similarly, subject 5 remarked on the difficulty of gene names, *“I can remember clopidogrel as an entire word, and CYP2C19, whatever the numbers are, I had a harder time keeping in my brain… I can remember a word much better than a random function of a gene.”* Finally, as subject 6 put it *“98 times out of 100, I get a couple of search terms that I can screen down, I can find out exactly what I want to go to very quickly.”*

### Time spent on case vignettes/ query entry

Median search time was 7 min per case (Table 3). During the search, subjects navigated to different pages or page sections using browser tabs and hyperlinks (median of 8.5 navigations per case). The median time interval between navigation events was 28 s. In 9 out of the 18 information-seeking sessions subjects entered search terms only once.

**Table 3:** Measures of information-seeking time and effort

Measurement	Range	Median	Average +/− sd
Information-seeking session duration (minutes)	2:41 to 15:08	7:14	8:22 +/− 3:57
Time between navigation events (minutes)	0:03 to 8:27	0:28	0:53 +/− 1:10
Number of page navigation events per subject per case	1 to 18	8.5	8 +/− 4.8
Number of Searches	1 to 8	1.5	2.3 +/− 1.9

## Discussion

We investigated physicians’ pharmacogenomics information-seeking behavior and needs through case vignettes. Study strengths include a mixed-method approach, rigorous thematic analysis of recorded information-seeking interactions and deepening interviews. Overall, the six physicians posed a wide variety of information needs and had significant challenges meeting these needs, spending quadruple, on average, the time clinicians will spend looking for information at the point of care [43] for general information needs. Thus, seeking pharmacogenomics guidance in today’s online resources may not be feasible for physicians. Our study findings provide design guidance for online resources to reduce barriers of effective use of pharmacogenomics in patient care decisions (summarized in Table 4). As an example, PharmGKB now provides an infobutton API that tries to provide a single summary page with information that covers touches on many of the themes we uncovered such as medications with known pharmacogenomics interactions, guidance on when to test, result interpretation, and therapy adjustment. Our results suggest that additional logistical information such as which test to order, how to order, cost and insurance coverage are also sought after. To support clinical decisions in pharmacogenomics, tools are needed which bridge content such as found on PharmGKB with logistical details for seamless navigation.

**Table 4:** Summary of design implications

Recommendation	Guidance
Facilitate berry-picking strategy	Provide multiple information berries in single content view. E.g. a bulleted synopsis of alternative treatments, evidence (including demographic data), indications, cost, other testing logistics
Facilitate berry-picking strategy	Guide user through information gathering process. E.g. provide basic pharmacogenomics information, such as the strength of clinical evidence, prior to providing logistical details
Provide clarity of sought after details	Unmask details which are hard to find and sought after. E.g. Clarify demographic prevalence data, and provide clear indications of alternative therapy that obviates the need for pharmacogenomic testing
Provide information on logistics	List laboratories offering test(s), provide test availability, highlight time to result, indicate insurance coverage and cost of test(s), provide a means to access an expert (either colleague who frequently uses the test or a pharmacogenomics expert)
Provide navigation support	Meaningful content headings and hyperlink labels.
Reduce navigation effort and short-term memory overload	Provide context specific information juxtaposed to relevant patient data

As expected, subjects sought expert consensus opinions and direct evidence, such as randomized clinical trials to support the clinical impact of genetic testing. In addition, the six physicians were interested in how genetic variants affect their patients’ health, such as phenotypic descriptions of test interpretations, similarly to Devine et al. [31]. This agrees with the finding that 80% of physicians would include information on genetic variation effects on drug activity in their ideal pharmacogenomic resource [44]. Less expected, subjects sought information to address knowledge gaps in genomics, such as the molecular basis for the clinical effect of gene variation.

In addition to clinical evidence, the six physicians questioned the logistics of carrying out a genetic test, including questions related to cost, availability, test turn-around time, insurance coverage, and accessibility of expert support. In general, this finding agrees with both a recent American Medical Association (AMA) survey [44] and a pharmacogenomics clinical decision support system study [31]. Interestingly, 64% of physicians in the AMA survey agreed that resources should include a list of the laboratories offering testing and indications of insurance coverage [44].

We also observed that physicians sought alternative approaches, which would obviate the need to consider genetic testing. It is possible that a sense of low self-efficacy and information overload may lead physicians to avoid genetic testing decisions. This is not unexpected, as ‘escape’ is a known strategy for dealing with information overload [18, 45] by physicians in other medical domains. The desire to escape is highlighted by the themes of ‘Prevalence of genetic variation ‘and ‘Indications for genetic testing.’ Subjects wanted to know if genetic testing was something they would need to worry about on a regular basis, in addition to tailoring care to their current patient. Interestingly, 77% of respondents of the AMA survey would include ‘demographics of populations likely to carry variations’ in their ideal information system [44]. Thus, delivering clear indications of the choice of an alternative therapy, along with clear demographic prevalence data may reduce search time.

On average, subjects navigated to new content eight times per case. Given the lack of experience most physicians have with pharmacogenomics, this finding is consistent with the ‘berry-picking’ information-seeking model, in which searchers ‘pick’ information from different locations, forming a better understanding of pharmacogenomics as they search [42]. In contrast, when physicians work within more general medical domains they rarely perform more than one search, look beyond one informational page or topic, and spend far less than 8 min looking for information [22, 24, 46, 47]. Similar behavior can be seen among medical students. In a previous study, more senior student’s retrieve specific summaries of information while younger students spent more time exploring basic information, to broaden their knowledge prior to meeting a specific need [48]. This may reflect the underlying ‘stability’ of the information need, which depends on the searcher’s expertise in the domain, as posited in a classical article by Ingwersen [49]. If the information need is ‘stable’ and ‘well-defined’ the information seeker will carry out ‘confined navigation’ directly targeted to meet their information need. For example, looking up quick reference information such as the dose and side effects of a drug. But in domains outside the searcher’s expertise, the information need is ‘variable, ’ leading to a more exploratory strategy such as berry-picking. Thus, the berry-picking strategy of our study subjects, consuming concepts to understand the relevance of facts, could be reflective of variable information needs given subjects inexperience in pharmacogenomics and need for basic understanding of the domain.

The berry-picking strategy could be facilitated by providing information berries in a single content view, rather than requiring users to navigate to multiple pages. Also, a user could be guided through the information gathering process by providing basic pharmacogenomics information, such as an indication of the level of clinical evidence, prior to providing logistical details. Additionally, structured search forms could help guide novice users through the search process, an approach that would be similar to PICO-based (Population, Intervention, Comparison, Outcome) searches employed in evidence-based medicine [50]. For example, a structured search form could include a choice to filter the results based on the information need (e.g., “when to test”, “what test to choose”, “interpretation of test results”, “logistics of testing”) and include an option to include in the results background information for non-experts in clinical genomics. Based on the order in which users are observed to use filters (i.e. exercises the berry picking strategy of iterating through information needs), the position of the filters in the search interface could be optimized to match the most common sequence of filter use.

Rather than refining search terms, subjects preferred navigational strategies such as hyperlinks. Thus, information resources could improve navigation support such as through the provision of meaningful content headings and hyperlink labels. The theory of information foraging, which premises that humans are ‘informavores’ seeking rich information patches [51], suggests that content headings and labels with strong ‘information scent’ are key to attracting users to the correct content.

Last, it is notable that subjects navigated back and forth between the case vignette and online resources. For design, this indicates that integrating online resource content with the patient context within electronic health record (EHR) systems [24] can reduce navigation effort and decrease short-term memory overload.

### Limitations

Time constraints were not imposed in the information-seeking sessions. Searching under typical clinical time constraints would likely affect search behavior. Yet, not imposing time constraints allowed us to observe a wide range of physicians’ information needs and the entire information-seeking process. In addition, due to the small sample size the quantitative measures are rough estimates. The case vignettes prompted study subjects to consider pharmacogenomics in their decision process. Similar ‘prompting’ might be needed in patient care contexts. Additionally, with six subjects we could not determine if thematic saturation was achieved; additional themes may emerge in a further study with a larger sample size, and expansion to a larger group would be useful in corroborating our results. Nevertheless, we believe this small number of subjects was sufficient to provide important insights that can help guide the design of pharmacogenomics information resources. Finally, we focused the subjects on the search for information within a specific resource and not on the choice of resource by constraining to UpToDate (all cases) and PharmGKB (for case 3).

## Conclusion

We observed the information needs and information-seeking behavior of six physicians when presented with three pharmacogenomics case vignettes. We found that physicians’ information needs included a need for phenotypic descriptions of test interpretations, a molecular basis for the clinical effect of drug variation, information on the logistics of carrying out a genetic test (including questions related to cost, availability, test turn-around time, insurance coverage, and accessibility of expert support), demographic prevalence data, evidence of authority for recommendations, and alternative therapies that would not require genetic testing. Physicians followed a pattern of information seeking consistent with the Berry Picking model, i.e. they followed iterative cycles of navigation and information collection which led to new information needs and additional knowledge exposure. We found that physicians searched an average of 8 min per case vignette, spent less than 30 s reviewing specific content, and rarely refined search terms. Further studies are needed to apply our study findings into information resources and assess their effect on pharmacogenomics’ practice.

## Additional files

Additional file 1: Case Vignettes. (DOCX 69 kb)

Additional file 2: Surveys used in the study. (DOCX 433 kb)

Additional file 3: Need Codes grouped by Theme. (DOCX 43 kb)

## Abbreviations

AMA: American Medical Association
ARTIC: Assessment by a double Randomisation of a Conventional antiplatelet strategy versus a monitoring-guided strategy for drug-eluting stent implantation and, of Treatment Interruption versus Continuation 1 year after stenting
CPIC: Clinical Pharmacogenetics Implementation Consortium
CYP2C19: Cytochrome P450 2C19
HPR: High on-treatment platelet reactivity
PharmGKB: Pharmacogenomics Knowledge Base
