# Supplementary material for: Physicians’ pharmacogenomics information needs and seeking behavior: a study with case vignettes
Source: BMC Med Inform Decis Mak. 2017 Aug 1;17:113. doi: 10.1186/s12911-017-0510-9 (PMC5540399; doi:10.1186/s12911-017-0510-9)

**Additional file 1: The following are the text/figures/links provided during the study.**

**Not a chart review. Please pretend you are in the moment described in the case.**

Case Study 1: Warm-up

Your patient is a healthy 28-year old woman of Ashkenazi Jewish descent. She and her husband, a Scandinavian, wish to have children in the future. Your patient's 10-year old sister was recently diagnosed with Gaucher’s disease (a recessive disease) and is being treated with Enzyme replacement therapy (ERT) using velaglucerase alfa. Your patient’s sister’s initial symptoms were splenomegaly, pain in her arms and chronic epistaxis. After normal liver function tests and hematocrit, X-ray and MRI showed signs of infiltration by macrophages. Thus, your patient’s sister’s doctor suspected Gaucher’s disease. A bilateral bone marrow biopsy showed clusters of enlarged cells with fibrillary cytoplasm, with crumpled tissue paper appearance, typical signs of Gaucher-cells. Your patient’s sister had no evidence of neurological problems. Finally, enzymatic assay for glucocerebrosidase was used to confirm a diagnosis of type 1 Gaucher’s disease.

Your patient would like to know what the chances are that she and her husband could have a child with Gaucher’s disease. Your patient tells you that her husband has been estranged from his family for years but remembers that his grandmother had some-kind of problem with an enlarged spleen. Your patient mentions that her mother has told her that there is a gene which causes Gaucher disease and that there is a genetic test which can help determine the benefit of using ERT. Both of your patient’s parents seem healthy. Considering the high cost of ERT, more than 400,000 dollars per year for a 70 kg patient receiving 60 U/kg every two weeks, your patient wants to be prepared.

As your begin telling your patient that you will refer her to a genetic counselor, your patient asks you if genetic testing might be necessary and what the gene is called.

***Please search*** [***UpToDate***](https://www-uptodate-com.ezproxy.lib.utah.edu/contents/search?forceHttps=true) ***till you feel confidant to manage the case and address your patient’s question on pharmacogenomics testing.

While you search please think aloud and highlight where you are looking. For example, you might say 'I'm looking to see who wrote Shakespeare's current play' while highlighting a link to 'Authors' in the text.***

Case Study 2

Sandy is a 7-year-old African male who has been prescribed both an inhaled corticosteroid (triamcinolone, Azmacort) and albuterol (salbutamol, ProAir HFA) to help control his persistent Asthma. His Azmacort metered dose inhaler delivers 200mcg/puff and is scheduled for twice daily use; his ProAir HFA metered dose inhaler delivers 90 mcg/puff, to be used as needed. His father has just scheduled an appointment at the Clinic because Sandy has had a week of sleepless nights due to increasing trouble breathing despite appropriate use of his inhalers including escalating use of albuterol. During the consultation you learn that his twin sister, who lives over 800 miles away in Seattle, has been prescribed equivalent medications and is experiencing similar difficulties with asthma control, including the need for an emergency room visit last month. Further, Sandy’s Father tells you that Sandy has needed to use the albuterol inhaler more than once a day for the past few months. Auscultation reveals inspiratory and expiratory wheezing which has worsened since Sandy’s initial visit a year ago. Follow up spirometric lung function testing reveals that his FEV1 has decreased from 96% to 80% of predicted for his age, weight and height since his last appointment six months ago.

In reviewing possible triggers for his asthma exacerbation, you note that the weather has been fair and that both air pollution and pollen counts have been minimal for the last month. Also, Sandy’s family does not have any pets, and his Father does not smoke, drink or use illicit drugs. Further, Sandy appears otherwise healthy, with no sign of viral infection. In short, there is no obvious trigger for Sandy’s respiratory difficulties. Finally, at the end of the consultation Sandy’s Father mentions that he saw a recent news report on the discovery that a gene alters the efficacy of asthma medications. He asks if pharmacogenomics testing is something that should be pursued.

During lunch, one of your colleagues shares with you how the result from a pharmacogenomics test allowed her to alter her patient’s treatment to avoid adverse drug events.This reminded you that last year at a conference on Asthma, a speaker discussed how certain inherited genetic variants are associated with worsening asthma symptoms in response to treatment with Albuterol. But you also recall being told that data on pharmacogenomics of Albuterol can be unclear.

***Please search*** [***UpToDate***](https://www-uptodate-com.ezproxy.lib.utah.edu/contents/search?forceHttps=true) ***till you feel confidant to manage the case and address the Father’s question on pharmacogenomics testing.***[***post-Case***](http://127.0.0.1/PostStudy.html)

Time Line


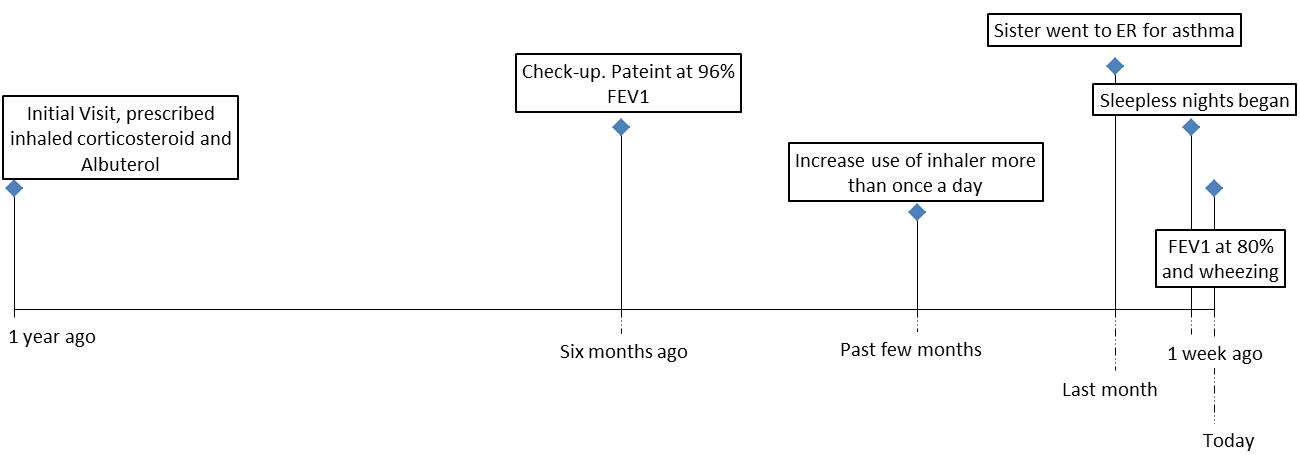


Case Study 3

Frank is a 64-year old male with coronary artery disease, who you are meeting in your office prior to planned coronary angiography/stenting (PCI) for angina symptoms. Having quit smoking at age 50, after 20 years at a pack a week, he seemed to be in excellent health. However, about two years ago he was diagnoses with angina and given a prescription for aspirin (75mg/day), simvastatin (40 mg/day), and a recommendation for lifestyle change. At that time he started an exercise regimen and reduced fat intake in his diet. Despite these lifestyle changes, six months ago he noted that episodes of chest pain increased to about once or twice per week precipitated by exercise or stress and relieved by rest. But recently the angina episodes have increased to several times per week. Last week a stress echocardiogram demonstrated anterior wall ischemia and angiography with possible PCI was recommended.

As per ACC guidelines, aspirin (75mg/day) will be continued and Frank has been prescribed clopidogrel (300 mg loading dose, followed by 75 mg/day). Reviewing his medical history there is no evidence of an increased risk of bleeding, nor is there a family history of increased clotting. During the consultation prior to the procedure, Frank mentions that his friend Sam was given a high dose of clopidogrel because Sam was determined to be resistant, due to a variation in CYP2C19 gene function. Frank asks you if he should also be tested for the genetic variant.

Frank’s question reminds you that in the morning of the consultation one of your colleagues was telling you about how variations in CYP2C19 can alter the metabolism of clopidogrel such that less active drug is present. You also recall reading about clopidogrel and pharmacogenomics testing, but you cannot quite remember all the details.

***Please search*** [***UpToDate, the CPIC clopidogrel summary from PharmGKB, and the clopidogrel CPIC guideline***](https://www-uptodate-com.ezproxy.lib.utah.edu/contents/search?forceHttps=true) ***till you feel confidant to manage the case and address Frank’s question on pharmacogenomics testing.***[***post-Case***](http://127.0.0.1/PostStudy_clopidogrel.html)

Time Line


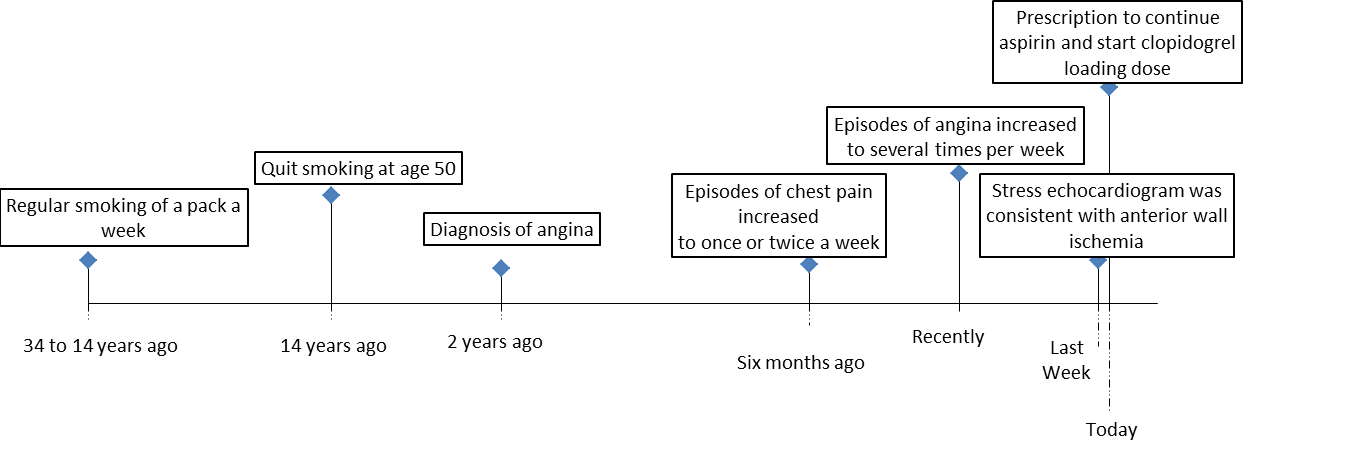

Supplement: Supplementary file 1 — Case Vignettes. (DOCX 69 kb) [file 12911_2017_510_MOESM1_ESM.docx]
