# Supplementary material for: Physicians’ pharmacogenomics information needs and seeking behavior: a study with case vignettes
Source: BMC Med Inform Decis Mak. 2017 Aug 1;17:113. doi: 10.1186/s12911-017-0510-9 (PMC5540399; doi:10.1186/s12911-017-0510-9)
Supplement: Supplementary file 2 — Surveys used in the study. (DOCX 433 kb) [file 12911_2017_510_MOESM2_ESM.docx]

**Additional file 2: The surveys used in the study are contained here. The pre-survey was used prior to the session, the post-surveys were used immediately after the indicated case.**

**-------------------------------------------------------**

**Pre-Survey**

Top of Form

1. How many years have you been in clinical practice (full or part time)?

2. What is your speciality (if any)?

3. What is your degree of comfort in using each of the following: (1=Very uncomfortable to 5=Very comfortable)

|  | Not familiar | 1 | 2 | 3 | 4 | 5 |
| --- | --- | --- | --- | --- | --- | --- |
| Internet |  |  |  |  |  |  |
| Word processing |  |  |  |  |  |  |
| Spreadsheet (Excel) |  |  |  |  |  |  |
| EHR |  |  |  |  |  |  |
| UpToDate |  |  |  |  |  |  |
| PharmGKB |  |  |  |  |  |  |

4. How frequently do you use each of the following (mark the box that reflects your usual practice):

|  | Never | Rarely | 1-4 per month | Weekly | Most days at work |
| --- | --- | --- | --- | --- | --- |
| Internet |  |  |  |  |  |
| Word processing |  |  |  |  |  |
| Spreadsheet (Excel) |  |  |  |  |  |
| EHR |  |  |  |  |  |
| UpToDate |  |  |  |  |  |
| Pubmed |  |  |  |  |  |
| PharmGKB |  |  |  |  |  |

5. Please rank the reasons that you use UpToDate or PharmGKB:

1= most common reason

2

3

4

5=least common reason

|  | UpToDate | PharmGKB |  |
| --- | --- | --- | --- |
| Help with clinical decisions |  |  |  |
| Keep abreast of evidence (regardless of patient) |  |  |  |
| Conduct research (formal studies) |  |  |  |
| Teaching |  |  |  |
| Find pharmacogenomic recommendations |  |  |  |

6. How frequently do you use each of the following for pharmacogenomics information (mark the box that reflects your usual practice): Use blank spaces to indicate other resources that you use

|  | Never | Rarely | 1-4 per month | Weekly | Most days at work |
| --- | --- | --- | --- | --- | --- |
| UpToDate |  |  |  |  |  |
| Pubmed |  |  |  |  |  |
| PharmGKB |  |  |  |  |  |
| DynaMed |  |  |  |  |  |
| Google |  |  |  |  |  |
|  |  |  |  |  |  |
|  |  |  |  |  |  |
|  |  |  |  |  |  |
|  |  |  |  |  |  |

**Following questions adapted from: National Pharmacogenomics Physician Survey 2008**

7. Was pharmacogenomics instruction included in your graduate medical education curriculum? Yes No

In your postgraduate medical education? Yes No

8. Do you believe that a patient's genetic profile may influence his/her response to drug therapy? Yes No

9. Do you feel that you are adequately informed about the availability of genetic testing and its application in the context of drug therapy?

Yes No

10. Do you rely on FDA-approved labeling (package inserts) for information regarding genetic testing to predict or improve the response to drugs? Yes No

11. Where do you obtain information on genetic testing and its application in the context of drug therapy? (select all that apply):

| Drug labeling (package insert) | Internet | Genetic testing laboratory |
| --- | --- | --- |
| Colleague/Other physician | Other |  |

12. At any time in the past 6 months, have you ordered or recommended a pharmacogenomic test for (select all that apply):

A patient? Yourself? A colleague or friend? A family member? None ordered or recommended

13. Within the past 6 months, with what average frequency have you ordered or recommended a pharmacogenomic test? (select one):

0 1 time/mo. 2-5 times/mo. >5 times/mo.

14. Pharmacogenomic tests have benefited your patients by (select all that apply):

| Improving drug effectiveness | Reducing drug toxicity | Increasing patients' understanding of their disease/therapy |
| --- | --- | --- |
| Improving patients' adherence to therapy | No tests ordered | Patients have not benefited |

15a. Do you anticipate ordering or recommending a pharmacogenomic test for a patient within the next 6 months? Yes No

15b. If you have not ordered or recommended a pharmacogenomic test in the past 6 months, or do not anticipate ordering one in the next 6 months, please indicate the main reason why (select one):

| Not applicable | Not enough knowledge about testing/genomic markers |
| --- | --- |
| Concern over privacy | Patients' resistance to genetic testing |
| Little-to-no or uncertain value in testing | I do not prescribe drugs with genomic tests available or recommended |
| Lack of insurance coverage for testing |  |

16. Are you more concerned about the loss of privacy of a patient's genetic information from the results of pharmacogenomic tests than from the results of other laboratory or diagnostic tests? Yes No

17. Do you believe that private, state, and federal health insurers should provide full coverage for pharmacogenomic tests? (select one):

Always Sometimes Never

18. What level of evidence is of importance to you in consideration of ordering a pharmacogenomic test? (select one for each of A through D below):

|  |  | **Very unimportant** | **Unimportant** | **Undecided** | **Important** | **Very important** |
| --- | --- | --- | --- | --- | --- | --- |
| A. | FDA approval or |  |  |  |  |  |
|  | recommendation |  |  |  |  |  |
| B. | Physician specialty |  |  |  |  |  |
|  | guideline |  |  |  |  |  |
| C. | Scientific journal |  |  |  |  |  |
|  | publication |  |  |  |  |  |
| D. | Recommendation or |  |  |  |  |  |
|  | experience of thought |  |  |  |  |  |
|  | leaders or respected colleagues |  |  |  |  |  |

19. A genome-wide scan, often available directly to consumers, is a scan of the patient's DNA that reveals markers associated with diseases. At any time in the past 6 months, have you ordered, recommended, or obtained a genome-wide scan or test for (select all that apply):

A patient? Yourself? A colleague or friend? A family member? None ordered or recommended

20. At any time in the past 6 months, has a patient brought into your office the results of a genome-wide scan obtained on his or her own Yes No

21. Your age: 20-29 30-39 40-49 50-59 60-69 ≥ 70

22. Gender: Male Female

23. Medical degree (select one): MD DO Other

24. Region of the US in which you practice (select one):

| Northeast (ME, NH, VT, MA, RI, CT, NY, PA, NJ) |
| --- |
| South (DE, MD, DC, VA,WV, NC, SC, GA, FL, KY, TN, MS, AL, OK, TX, AR, LA, PR, US Virgin Islands) |
| Midwest (WI, MI, IL, IN, OH, ND, SD, NE, KS, MN, IA, MO) |
| West (ID, MT,WY, NV, UT, CO, AZ, NM, AK,WA, OR, CA, HI, Pacific Islands) |

25. How would you describe your practice setting (select one): Urban Suburban Rural

26. Location of medical school attended (select one): US Europe Other

27. Number of years since your medical school graduation (select one):

< 5 5-9 10-14 15-19 20-24 25-29 30-34 35-39 40-44 ≥ 45

28. What is your primary practice speciality? (select only one)

| Allergy/Immunology | Family/General practice | Ophthalmology | Plastic surgery |
| --- | --- | --- | --- |
| Anesthesiology | Internal medicine | Orthopedics | Preventive medicine |
| Cardiology | Medical genetics | Otolaryngology | Psychiatry |
| Dermatology | Neurology | Pathology | Radiology |
| Emergency medicine | Neurosurgery | Pediatrics | Surgery |
| Endocrinology/ | Obstetrics/Gynecology | Physical medicine | Urology |
| Diabetes/Metabolism | Oncology | & rehab | Other |

29. Primary employer (select one):

| Self-employed solo practitioner | Private group practice | Hospital | Medical school/University | Military |
| --- | --- | --- | --- | --- |
| HMO or other health insurer | State government | Federal government | Veteran's Administration | Other |

30. What is the average number of patient visits you conduct per day? (select one): None 1-9 10-20 >20

31. Who is the primary insurance carrier for the majority of your patients (select one):

None Private insurance Medicare Medicaid VA Military/Tricare

Bottom of Form

# Post-Session Case 2

### please do not press enter till finished

Top of Form

1. What is your perceived complexity of the vignette? (1=least complex; 5=most complex)

2. What is your experience managing patients like the one in the) vignette? (1=least experience; 5=most experience

3. What is your final answer to the case vignette?

4. Could you please summarize in 1-2 sentences the gist of the evidence that guided your decision?

5. What other types of information could have helped you? (What additional information do you think would be useful to resolve the issues in this case?)

6. The information I found1=strongly disagree5=strongly agree

|  | 1 | 2 | 3 | 4 | 5 |
| --- | --- | --- | --- | --- | --- |
| Enhanced my decision-making |  |  |  |  |  |
| Increased my knowledge |  |  |  |  |  |
| Helped me recall something I had forgotten |  |  |  |  |  |
| Increased my level of uncertainty |  |  |  |  |  |
| Frustrated me with the information-seeking process |  |  |  |  |  |
| Increased my confidence in making the right decision |  |  |  |  |  |
| Improved my comfort in managing this patient |  |  |  |  |  |
| Made me more likely to refer this patient to a specialist |  |  |  |  |  |
| Surprised me |  |  |  |  |  |
| Took significant effort scanning / skimming information |  |  |  |  |  |

7. Rate the following types of information found for this vignette (1=Not at all; 5=A great deal; NA = not applicable):

|  | Helped with my decision | Updates my knowledge | Required significant effort scanning / skimming |
| --- | --- | --- | --- |
| UpToDate |  |  |  |

Bottom of Form

# Post-Session Case 3

### please do not press enter till finished

Top of Form

1. What is your perceived complexity of the vignette? (1=least complex; 5=most complex)

2. What is your experience managing patients like the one in the) vignette? (1=least experience; 5=most experience

3. What is your final answer to the case vignette?

4. Could you please summarize in 1-2 sentences the gist of the evidence that guided your decision?

5. What other types of information could have helped you? (What additional information do you think would be useful to resolve the issues in this case?)

6. The information I found1=strongly disagree5=strongly agree

|  | 1 | 2 | 3 | 4 | 5 |
| --- | --- | --- | --- | --- | --- |
| Enhanced my decision-making |  |  |  |  |  |
| Increased my knowledge |  |  |  |  |  |
| Helped me recall something I had forgotten |  |  |  |  |  |
| Increased my level of uncertainty |  |  |  |  |  |
| Frustrated me with the information-seeking process |  |  |  |  |  |
| Increased my confidence in making the right decision |  |  |  |  |  |
| Improved my comfort in managing this patient |  |  |  |  |  |
| Made me more likely to refer this patient to a specialist |  |  |  |  |  |
| Surprised me |  |  |  |  |  |
| Took significant effort scanning / skimming information |  |  |  |  |  |

7. Rate the following types of information found for this vignette (1=Not at all; 5=A great deal; NA = not applicable):

|  | Helped with my decision | Updates my knowledge | Required significant effort scanning / skimming |
| --- | --- | --- | --- |
| PharmGKB |  |  |  |
| UpToDate |  |  |  |

Bottom of Form
