# Supplementary material for: Physicians’ pharmacogenomics information needs and seeking behavior: a study with case vignettes
Source: BMC Med Inform Decis Mak. 2017 Aug 1;17:113. doi: 10.1186/s12911-017-0510-9 (PMC5540399; doi:10.1186/s12911-017-0510-9)
Supplement: Supplementary file 3 — Need Codes grouped by Theme. (DOCX 43 kb) [file 12911_2017_510_MOESM3_ESM.docx]

| **Additional file 3. Need Codes grouped by Theme** These are the information Needs identified in the paper with examples of raw quotes. They are arranged by Theme. | | |
| --- | --- | --- |
| Code (themes in orange) | Code definition | Quote examples |
| **Alternative Therapy options** |  |  |
| Need: alternative treatment option | Need evidence and recommendation for alternative treatment, without having tested yet. i.e. Is there an alternative treatment recommended which avoids needing to consider a genetic test? | Subj 5 "...telling you to just be using another option in him or does he have a particular contraindication to just doing alternate therapy. But you know I’m supposed to pick up on instead of doing any testing on him. " |
| **Role of genetics in the manifestation of the disease** |  |  |
| Need: effect of genetic variation on worsening disease (not treatment failure) | Need for information on genetics of the disease being treated. | "I want to know about the genetic variant effects that are associated with worsening asthma symptoms" |
| D- Need: evidence for effect of Genetics on disease prognosis | A need to know if there is a link between genetic variation and risk of worsening of the disease | "I was looking for very specific reference or references that might have said that there was a genetic predisposition to worsening symptoms. What I should have been thinking at the same time was also that albuterol played a role in it." |
| Disease genetics - the first case is about potential kids (patient who might exist) | Need understanding of disease genetics. What variants are associated with which disease phenotypes or sub-types of disease |  |
| Need: Disease genetics | Need understanding of disease genetics. What variants are associated with which disease phenotypes or sub-types of disease | "And we did find a few references...That, that I could have looked up that suggested that, that the presence of that variant may, you know, did have a role in asthma exacerbation. So there are probably you know are genetic variants linked to prevalence of asthma, which it leads to the, I don’t find that. That I didn’t find." |
| **Clinical Impact of Genetic Testing** |  |  |
| Need: effect of genetic test result on treatment course (how to) | What is the change in treatment course if the variation is found? Need information that points to the value of genetic testing - major changes in treatment course | "…so, they're suggesting another drug for ultrametabolizers" |
| Need: management of treatment failure | Need information on how to manage treatment failure | "…I'm looking for something…oh, management criteria..." |
| Need: effect of genetic variation on treatment efficacy | Need to know how treatment failure, or reduced treatment efficacy, due to genetic variation manifests itself. This is the question of 'what does a problem look like?' | subject 3: starts with search for 'pharmacogenetics asthma', looks at section 'Overview of pharmacogenomics' and says ' reading through sections on management of treatment failure, hovers over 'Assessment of exacerbation severity' within 'Acute asthma exacerbations in children: Home/office management and severity assessment' and states title. … subject 3 excited "okay, wheezing phenotypes prediction of asthma, asthma risk factors predictive tools...<clicks on Asthma Risk Factors> I'm not sure if this is going to get us...I'm still searching for something that will help me, with testing that might lead to genetic aspects of worsening" <transitioning to next need> Next quote; subject 3 near 4:12. subject voices need and then we have the search term of 'pharmacogenetics asthma' to end the evidence 'I don't see getting me to what I need for pharmacogenetics, that might tell me about genetic aspects of worsening' Next quote: 'effect of genetics, relation to treatment' subject 1 10:40, Next quote: Subject 6 at 27:00 ' I was trying to figure out the relevance of high platelet reactivity' interview notes next quote: subject 1 around 0:28:45.000 'I like this failure thing because I wonder if that might have something to do...if the genetics might have something to do with failure' |
| Need: evidence for not doing genetic testing | Need to find and understand evidence that recommends not performing a genetic test. |  |
| Need: genetic disease diagnosis | Need to understand the manifestation of the phenotype of the disease that are used in diagnosis | <audio/video quote> subject comments on looking for the right information ("labs") and is collection info-berries on diagnosis of Gaucher's disease "sometimes I can see something that will point me to initial lab testing and diagnosis" |
| Need: Strong Recommendation | Needing genotypic classification suggesting a phenotype that has relevance to your therapeutic intervention | from interview "ah, they were more confident in the, the evidence base than you know the final recommendations, the summary recommendations in the UpToDate. Meaning that um, UpToDate suggested ah, at least based on my reading that um, that ah, ah, genotypic testing is in most cases um, not helpful or difficult to interpret. Whereas here I’m seeing you know recommendations that are strong, strong...Um, you know based on ah, a genotypic classification suggesting a well a, a phenotype that has relevance to your therapeutic intervention." |
| Need: significance of the effect of genetic variation on medication efficacy | Need to know what signs of treatment failure due to the effect of the variant on medication efficacy. How does the variant’s effect on medication efficacy 'show' itself in the patient? This is not the need for knowing only if treatment failure can happen in the patients with the genetic variant. Here the need is for a description of the manifestation of the effect of the genetic variation on treatment efficacy. | "you've got clopidogrel resistance and treatment failure" inside the overview of pharmacogenomics” and subject 2 around 14:40 #00:07:30-1#  Okay. What happened there? Resp: So, I wanted to look at what information there was about patients with asthma and albuterol and how their genetic profile affects albuterol. And I just wasn’t seeing a search that was really jumping out of me at the time that was getting, That felt was a high yield link to finding information that I was looking for.  #00:08:01-9#  So at that point, I just was going to click on it and start skimming through the theme, because I wasn’t seeing anything that just definitively jumped out and said, “Oh here is the information that you need.” Intvr: What would it have looked like? Resp: So, I was hoping that I would see something like ah, albuterol failure in asthma or ah, issues associated with treatment or treatment failure in asthma or something like that. And then when I clicked on that link there was a sub tab like genetic issues or genetic variance or something like that.  #00:08:31-3#  Intvr: And what then what were you expecting to see when you click if that link was there, if you clicked on it what would you see? Resp: So I would, I would expect to see something about some clinical trial that had been performed looking at albuterol failure in patients and was found it was associated with certain genetic profile. Or there being case studies of patients who, who, who do not um, who do, who are what’s the word I’m looking for? Um, who failed albuterol treatments based upon an underlying genetic variant?" |
| **Understanding General Molecular effect of genetic variant** |  |  |
| d -need: phenotype expected from genetic variant (is the molecular change likely to be significant enough to impact patient phenotype) | Need to know effect of mutation on protein activity (i.e. the variant is a nonsense mutation which produces a truncated protein that has zero function). This is a need for understanding how genetic variation leads to phenotype. | in same place "Just that there was a, you know a handful of mutations that account for nearly all of the, all the disease there.” This is information that was important, but we did not code the information they found “trying to recall my molecular biology.  #00:26:01-2#   And ah, you know what is a mis, missense mutation" |
| **Specific, actionable, clinical guidance from authoritative sources** |  |  |
| Need: authoritative direct guideline for testing | Need a trusted statement that a test would be done to decide the course of care. Which Authority made the statement about pharmacogenomics? | "with the authorities, with the experts, with the review of the literature American Heart Association and the rest " |
| Need: summary of recommendations | Needing a conclusive recommendation (with authority) on when testing should be done (similar to other codes). (a statement that is direct) | subj3 case3 16:00 "was going for a bottom-line here" on CIPIC guideline. Clinically actionable statement from authoritative source. |
| Need: Summary from specialist (e.g. genetic counselor) | Summary of from a specialist on genetic information specific to the case | Audio/visual subject "…genetic counselor…" and proceeds to collect information from the section. The evidence is location, information and some verbalization |
| **Guidance on optimal approach to genetic testing** |  |  |
| need: specific test to use | Looking for mention of a specific test to use. This can be any diagnostic test, from metabolic test to a test for carrier status. | "I'm not finding anything that is like a straightforward test…"(subject3), "this is what we are talking about…this test…"(subject 3 case 3 near 7:00 #00:35:30-4# not a great quote), "Again I think looking for if there were any, if, if there was any indication that I should select a particular genetic test option"(subject 5) |
| need: testing recommendation (specific variant) | Should a specific genetic variant be tested for? |  |
| D-Need: clear guidance on interpretation | Need to know important caveats to a particular test that effect interpretation | "genotyping may miss that ah, some of those loss of function alleles. And um, I just find it interesting that, that’s it’s a, it’s a footnote because it seems rather important to me. " (subject was looking at CPIC interpretation table) |
| **Help with search terms** |  |  |
| Need: Correct spelling of gene name... | Need for correct spelling of search term | subject 4 copying and pasting gene name |
| Need: alternative search criteria | Need for term synonyms when searching for information | "word is easier to remember than a function or a name…" |
| D-Need: medication specific detail | Need more information on medication - drug class, mechanism (molecular details) - to aid in finding genomic information | subject 6"but I should have put in β2-agonist in front of pharmacogenomics or something like that. “ “Basically confirming that this Salbutamol was a short-acting β2-agonist.”<"So you know, I got asthma at the pharmacogenomics and 98 times out of 100, I get a couple of search terms that I can screen down, I can find out exactly what I want to go to very quickly."> |
| **Indications for genetic testing** |  |  |
| Need: The characteristics of patient for whom testing is recommended | What are the characteristics of patient's that need testing? Can be familial, phenotypes, clinical course to date | Subject 3: some instance group where you were to tell me that that gentleman needed to be tested before he had a stent put in. That’s what I was searching for. Also found in the interview of subject5 "the information that I was looking for the whole time that I didn’t feel like I really found in a really concentrated way, was here are the risk factors that you as the clinician want to be looking for in your own patient. That is going to send you over the edge to actually get genetic testing. "Subject 5 "A bulleted list that says risk factors for testing." Subject 5 at 35:20 "data on the selected patient population" and in deepening: And then you are going to go to select data. <<35:41(subject has highlighted ‘select population populations’ in the CPIC guidelines) >>  #00:20:54-5#   What were you, what did you want from that? Resp: Same thing as that I wanted the whole time. Intvr: Okay. Resp: Just what, what is this selected patient population and I don’t feel that I know what people of the selected data population is. |
| D-Need: family history | A specific need for the family history of the patient. |  |
| **Prevalence of genetic variation** |  |  |
| Need: prevalence of disease in specific population | How frequent is the phenotype in the patient's population? In order to determine risk. | Subject 6 Resp: Yeah, so I was just reading up the various ethnic groups and I didn’t see Scandinavians in there. Intvr: Okay. Resp: But of course there are Ashkenazi ethnicities that live in Scandinavia.  Intvr: So, so this is ethnicity that’s how you would classify this information that you are looking for information that is targeting the patient’s ethnicity.  #00:19:30-4#  Resp: Exactly that’s right. So like, yeah, if, if more people from ah, from Northern Europe ah, are more likely to harbor ah, ah, you know be heterozygous for Gaucher’ s. |
| Need: prevalence of important test result | The occurrence rate of test results that require action | Subject 4 "it doesn't really tell me what percentage of the population has an issue with CYP2c19". Subject 6 "…just how many individuals do I need to test before I find…individuals who either metabolize rapidly or poorly." |
| NEED: prevalence of specific gene mutation | Need to know the prevalence of a variant |  |
| D-Need: inheritance pattern to inform whether family history is important | How is the phenotype inherited? How penetrant is the mutation? (dosage details) And is there family history of phenotype? | 37:30 subject 4: And this was at the very end 38, 34 or so. And you um, highlighted autosomal co-dominant, is that important? Resp: It is important because if you have a purely autosomal dominant effect, then you would expect that someone in the family would have had the issue at some point in the past. They probably would be in the clopidogrel was a new medication and the knowledge about the Cytochromes P450 mutations is also rather new.  #00:01:03-5#  And so you may or may not actually get information from the family history about an issue. But if there had been a pure autosomal dominant, maybe you would actually have a family history of something that would be relevant.  But the co-dominant is not quite as important, because you may have people who have a minor issue, but they just don’t know about it and it’s only if you are lucky enough to get both copies of the gene that you would actually have a true issue.  #00:01:32-1#  Intvr: Great thank you. Resp: But even then it decreases the chance that they going to have an issue with it, the co-dominance. ‘Cause you need to get 2 parents who have that co-dominant gene. Intvr: So for a pharmacogenomics relationship then, you would want to know the, the type of variation. Does it cause an autosomal auto, sorry is it a co-dominance or is it a? Resp: Is it recessive as a co-dominant, is it a true dominant. |
| D-need: relative cost of genetic testing compared to disease prevalence | How much does the test cost? Is the problem prevalence enough to warrant the cost? |  |
| **Logistics of testing** |  |  |
| D-need: genetic test turnaround time | Will the test result/interpretation arrive in time to make a difference? | subject3 case3 Resp: But can they turn this around? <new d-need> Intvr: I like that question. So yeah I was still going to still ask you about that so. Resp: ‘Because you had, you know well you would have to know I mean if the guy is going to have the stent the next day.  #00:15:31-5#  Intvr: Yeah. Resp: Can, can you get his testing this type of test in half an hour with DOT-Blot-PCR. Intvr: Yeah. Resp: And tell you yeah he has got the variant - he doesn’t. He has this variant, this variant no he does not. And yeah, they can do that stuff they just do a little you know well you know more about this than me. They have the DNA sequence there and they have the little dot-blot and they can see it. |
| *SOME COST QUOTES* | Quotes related to subjects thoughts on genetic test costs. | DURING POST CASE Subject 4 (44:00) Other information that could have helped me? Ah, knowing the prevalence of the…   #00:44:00-4#  …CYP2C19 mutations in the general population and in subset populations, knowing if the patient had had family members with known…  #00:44:30-4#  …difficulty in metabolizing medications, cost of the test, availability of the test, turnaround time of the test. Subject 4 deepening Resp: I think that it definitely if you have a test that costs $500,000 to do, then most of the time I’m going to say no it’s not even remotely worthwhile, unless it’s going to potential millions have a test for us to do, tells you are congested. Intvr: Where is that kind of information?  #00:11:30-8#  Resp: Because I haven’t really seen it before. Intvr: Or any diagnostic test is that right? Resp: Um, pretty much for any diagnostics test. My experience is that people who allow the test to happen in the hospital systems, they don’t really want that information to be available to the people who are ordering the test? Intvr: Would have been great to have some sort of information because it’s expensive. Resp: We can have a ballpark. Yeah, ballpark to say here potentially they make sure and the downside to get this kind of benefit. Intvr: Or maybe um, would it be useful if they said, this costs 1/3 of an MRI or a hospital this kind of involved in that kind of disposal to give you. Resp: This gives you a summarization of information, MRIs has got every single detail.  Intvr: Yeah, sure. Resp: But at least that type of information that tells you that it’s in the range of hundreds as opposed to thousands, as opposed to hundreds of thousands. |
| D-Need: how soon will a test be available for ordering | How soon will the test be available to order in my system? | subject 4::Intvr: Um, any questions for me? Resp: No, other than how fast can we make the genomic test actually be available? :: subject 4 “subject 4 near 4:00 ‘first I’d have to find out if it even exists and how much it costs’” |
| **Practice changing evidence** |  |  |
| Need: effect of Genetics on medication efficacy | Need to know if genetic variation disrupts efficacy of a specific medication in treating a specific disease. The question of 'Is there a problem?' | “potential site of mutation that I can look for to see if there is an issue of metabolizing that particular medication."  NEXT QUOTE: case 3 num2. @07:15 in section on Variation in clopidorel metabolism "I'm a little bit confused now, just thinking out loud, as to how the variations of this gene, whether it increase the dug being around or decreases drug, I'm not particularly clear on that issue" |
| Need: Genetic testing recommendation | Need a recommendation on whether or not testing should be performed when considering using the medication to treat the disease under consideration. | "whether genetic testing could be or should be taken into account. I know and hoping that they had this, a particular. Again should you order the study or should you order for the test" and "…hopeful that that meant the guideline updates were down there somewhere that directed the actual testing recommendations" AND " Should we test patients for the mutation when we start off, what are their recommendations?" subj6 "this group suggests that there is benefit" subj5 "hopeful that that meant the guideline updates were down there somewhere that directed the actual testing recommendations." |
| Need: evidence for effect of Genetics on medication efficacy | Need to have strong/clear evidence that links phenotype associated with adverse events or medication failure with genetics (strong evidence that the phenotype is linked to genetic variation) | subject 2" I was looking here at a randomized clinical control trial looking at the use of prasugrel and clopidogrel and the prevalence ...Then on the RAPID GENE study 200 patients undergoing PCI were randomly assigned to either a rapid point-of-care genotyping for the CYP2C allele given 10mg and 5mg of clopidogrel...So essentially this information is telling me is the patients with the C, CYP2C19 mutation prasugrel is often better and that is what I was looking for.":: and "So this was a more, so I was looking for very specific reference or references " ::and **"But um, essentially you know the, the um, whenever you know clinical literature says um, data suggests or um, maybe relevant that sort of thing, um, you realize that the evidence basis is still um, in its nascence. It may still be weak. "** |
| Need: screening recommendations | Need for recommendation on screening for pharmacogenomics variant as part of routine clinical course. This differs from a testing recommendation as this is not a qualified recommendation. In other words, the need is to learn if there is a recommendation for routine screening in all patients. | Subject 5 near 29:20. is highlight with mouse and stating 'almost 2500 patients' while looking at the ARTIC-monitoring trial. In screening recommendations. Also reads 'do not recommend routine testing' |
| Need: evidence for treatment decisions based on pharmacogenomics | Need to have strong/clear evidence for decision on pharmacogenomics decisions | subject 3 near 8:40 "it is not completely clear in my mind yet about the evidence for doing the testing for the variants’…how standard that is and how clear that is…here is genetic testing, ideally we should find whether or not we are supposed to do variant testing" subject 6 Resp: Ah, I, I liked that it was you know for, for the clinical question we had it was um, simpler and more succinct than, than UpToDate. Um, but it seemed that…  #00:07:30-2# …ah, they were more confident in the, the evidence base than you know the final recommendations, the summary recommendations in the UpToDate. Meaning that um, UpToDate suggested ah, at least based on my reading that um, that ah, ah, genotypic testing is in most cases um, not helpful or difficult to interpret. Whereas here I’m seeing you know recommendations that are strong, strong. |
| Need: evidence for not doing genetic testing | Need to find and understand evidence that recommends not performing a genetic test. |  |
| D- Need: Summary and recommendations | Need a bottom-line, i.e. brief synopsis with a recommendation | Subject 3 code is listed as deepening but should be regular code! |
| D - Need: practice based information (guidelines) | Need a recommendation that contains specific actions | Subject 1" the summary did not have any recommendations. it had a summary of the data but the data was too far removed from actually me being able to take one step or another. " |
| D - Need: population based evidence specific to patient | How often has the patient demographic been studied in relation to the variants’ effects? In other words, has research been done on patients of my patient's demographic? | With patient 1 "if they are unrepresented in the studies then how am I going to know how I am going to interpret this. I am liable to come back with something that says we do not have enough information" and "S: So, it’s not that it made up my mind, it’s just that in clinical practice I am weighing hundreds of different things B: yes S: and I'm putting them on two sides of should I or should I not do it. This is a little thing on the side of nah. if it was... 07:25 S: for instance...if we were back...if we were looking at a test for a particular disease, like sickle-cell, and I've got a somebody whose African American, or someone from the Mediterranean area B:yep S: and I'm thinking that's the population. I am going to be much more likely to do it, because that's the population that's been studied. Does that make sense? So if it’s not in the right population...if the studies aren't there...it’s not that I'm going to not do it...I am going...you know, do I have other things to do first. |
| D- Need: effect of genetic test results on expectation of the efficacy of treatment | How often does using the test result improve outcomes? | "Then on the RAPID GENE study 200 patients undergoing PCI were randomly assigned to either a rapid point-of-care genotyping for the CYP2C allele given..." |
